# Anæmia and Some of the Diseases of the Blood-Forming Organs and Ductless Glands

**Published:** 1900-03

**Authors:** 


**Anæmia and Some of the Diseases of the Blood-forming Organs and Ductless Glands.** By BYROM BRAMWELL, M.D., F.R.S. Ed. Pp. 450. Edinburgh: Oliver and Boyd. 1899.

There is very little new in this book, but it presents detailed and accurate accounts of a most interesting group of diseases, which it is most convenient to have in one volume. The style is rather that of the lecture room—too easy and redundant—than that of a book to be read; and there is some amount of repetition. The volume contains descriptions of the various kinds of anæmia, of leucocythemia, Hodgkin's disease, Addison's disease, myxœdema, exophthalmic goitre, and acromegaly; but there are some omissions to be noted: there is no mention of the parathyroid glands and of the possible part they play in exophthalmic goitre, nor of the rare disease achondroplasia, so closely related to cretinism, and Hutchison's researches on the chemistry of the thyroid gland are not described.

The attempts to harmonise conflicting views are sometimes amusing. Thus, in discussing the etiology of Addison's disease, Dr. Bramwell states that "some of the symptoms . . . are due to destruction of the [suprarenal] capsules, while others are due to implication of the nervous structures with which they are connected," but does not make the least attempt to state what symptoms are referable to each of these lesions.

One great defect of the book is the absence of illustrations. For these one must turn to the author's excellent *Atlas of Clinical Medicine*. The book under review is in reality made up of articles selected from the *Atlas*, amplified and brought abreast of current knowledge, but without the pictures which gave to the former work such lasting value.

